# Comparison of enhancement quantification from virtual unenhanced images to true unenhanced images in multiphase renal Dual‐Energy computed tomography: A phantom study

**DOI:** 10.1002/acm2.12685

**Published:** 2019-08-19

**Authors:** D. Olivia Popnoe, Chaan S. Ng, Shouhao Zhou, S. Cheenu Kappadath, Tinsu Pan, A. Kyle Jones

**Affiliations:** ^1^ MD Anderson Cancer Center UT Health Graduate School of Biomedical Sciences Houston Texas; ^2^ Department of Diagnostic Radiology MD Anderson Cancer Center Houston Texas; ^3^ Department of Biostatistics MD Anderson Cancer Center Houston Texas; ^4^ Department of Imaging Physics MD Anderson Cancer Center Houston Texas; ^5^ Present address: Department of Imaging Physics, Medical and Radiation Physics, Inc. San Antonio Texas

**Keywords:** Dual‐Energy CT, virtual unenhanced imaging, renal DECT

## Abstract

Multiphase computed tomography (CT) exams are a commonly used imaging technique for the diagnosis of renal lesions and involve the acquisition of a true unenhanced (TUE) series followed by one or more postcontrast series. The difference in CT number of the mass in pre‐ and postcontrast images is used to quantify enhancement, which is an important criterion used for diagnosis. This study sought to assess the feasibility of replacing TUE images with virtual unenhanced (VUE) images derived from Dual‐Energy CT datasets in renal CT exams. Eliminating TUE image acquisition could reduce patient dose and improve clinical efficiency. A rapid kVp‐switching CT scanner was used to assess enhancement accuracy when using VUE compared to TUE images as the baseline for enhancement calculations across a wide range of clinical scenarios simulated in a phantom study. Three phantoms were constructed to simulate small, medium, and large patients, each with varying lesion size and location. Nonenhancing cystic lesions were simulated using distilled water. Intermediate (10‐20 HU [Hounsfield units]) and positively enhancing masses (≥20 HU) were simulated by filling the spherical inserts in each phantom with varied levels of iodinated contrast mixed with a blood surrogate. The results were analyzed using Bayesian hierarchical models. Posterior probabilities were used to classify enhancement measured using VUE compared to TUE images as significantly less, not significantly different, or significantly higher. Enhancement measured using TUE images was considered the ground truth in this study. For simulation of nonenhancing renal lesions, enhancement values were not significantly different when using VUE versus TUE images, with posterior probabilities ranging from 0.23‐0.56 across all phantom sizes and an associated specificity of 100%. However, for simulation of intermediate and positively enhancing lesions significant differences were observed, with posterior probabilities < 0.05, indicating significantly lower measured enhancement when using VUE versus TUE images. Positively enhancing masses were categorized accurately, with a sensitivity of 91.2%, when using VUE images as the baseline. For all scenarios where iodine was present, VUE‐based enhancement measurements classified lesions with a sensitivity of 43.2%, a specificity of 100%, and an accuracy of 78.1%. Enhancement calculated using VUE images proved to be feasible for classifying nonenhancing and highly enhancing lesions. However, differences in measured enhancement for simulation of intermediately enhancing lesions demonstrated that replacement of TUE with VUE images may not be advisable for renal CT exams.

## 1 INTRODUCTION

Multiphase computed tomography (CT) exams are a noninvasive imaging technique commonly used for the diagnosis of renal masses.^1^ These exams include a true unenhanced (TUE) phase of imaging, the administration of an iodinated contrast agent, followed by one or more postcontrast phases. Enhancement can be quantified by calculating the difference in CT number between TUE and postcontrast images for a given region of interest (ROI). It is crucial that the quantification of enhancement be accurate, as it has been characterized as the most important criteria in determining surgical from nonsurgical renal masses.^2^ Previously, a change in CT number of 10 Hounsfield units (HU) or more between pre‐ and postcontrast images was considered positive for enhancement; however, with the advent of helical CT it has been proposed that this threshold should be increased to account for helical interpolation.^3^ Enhancement is now commonly characterized by a change of 20 or more in measured CT number between TUE and postcontrast images, although this number is not universally agreed upon.^2^ As a result, a mass with enhancement measuring between 10‐20 HU can be considered “intermediate” and may require further evaluation.^2^

Renal cell carcinoma (RCC) is the most common kidney cancer in adults, accounting for approximately 90% of renal neoplasms and 3% of all adult malignancies.^4^ RCC is an aggressive disease that has a 5‐year survival rate of 95% for Stage 1 disease, but less than 20% for Stage 4 disease.^5^ The diagnosis of RCC based on the appearance of a lesion on CT imaging can vary widely in difficulty. While the diagnosis of a simple nonenhancing cyst is straightforward, classifying complex lesions can be much more challenging.^1^ Studies have shown that if the patient has an enhancing renal mass, such as RCC, the mass will have a substantial noncalcified region with a CT number measuring within a range of 20‐70 HU on unenhanced CT.^6^ In a postcontrast scan acquired during the corticomedullary phase, studies have shown that RCC will enhance significantly more than a benign cyst (81.4 HU vs 27.4 HU, respectively) and that a difference of >42 HU in measured enhancement during the corticomedullary phase was highly predictive of RCC with 97.1% sensitivity and 85.7% specificity.^7^

Dual‐Energy CT (DECT) is an extension of conventional CT in which two datasets are acquired using different photon spectra nearly simultaneously.^8^ This can be achieved either by using a single X‐ray tube that rapidly switches between a high and low kVp at each projection angle, scanning the patient twice using different kVp, scanning the patient with dual X‐ray sources and detector arrays, or using a dual‐layer detector with a single X‐ray source. This work uses the rapid kV‐switching technique in which the X‐ray tube alternates between 80 and 140 kVp at each projection with a constant tube current of approximately 600 mA to acquire co‐registered dual‐energy projections.^9^ Benefits of rapid kV switching include excellent temporal registration, which reduces the potential for motion artifacts, and the availability of the entire scan field of view (SFOV) for DECT image acquisition.^10^ A technical challenge of this technique is the rise and fall times of the high voltage waveforms, which complicates the determination of the effective energy for the high‐ and low‐kVp projections.^11^

DECT provides the ability to exploit the attenuation properties of materials to apply material decomposition techniques. This is achievable because each material has a unique attenuation coefficient, based on a unique combination of Compton and photoelectric interaction probabilities. A basis pair of materials with a large separation in linear attenuation coefficients can be chosen, commonly water and iodine, and used for material decomposition. By assuming each voxel is a weighted combination of the basis pair, the amount of iodine in each voxel can be estimated when the object is imaged at different energies. Theoretically, material decomposition can be generalized to decompose an arbitrary number of materials^12^; however, this work focuses on basis pair decomposition. Material decomposition is the basis for the reconstruction of virtual unenhanced (VUE) images, in which the estimated volume of iodine in each voxel is replaced by an equivalent volume of blood.^13^

The use of VUE imaging provides the potential to use VUE images in place of TUE images in multiphase renal CT exams. Eliminating the precontrast phase of imaging could reduce patient dose and increase patient throughput, consequently improving clinical efficiency. Previous studies have investigated the feasibility of using VUE images in place of TUE images for patients with gastric tumors, resulting in a dose reduction of 30.5%, and in the diagnosis of patients with subarachnoid haemorrhage.^14^, ^15^ For imaging of renal lesions, it has been shown that a threshold of 2 mg/cm^3^ is the most accurate in distinguishing enhancing from nonenhancing lesions using iodine density images generated from DECT.^16^ Other studies have investigated the feasibility of replacing precontrast images with virtual noncontrast images in renal DECT exams.^8^, ^17^, ^18^, ^19^ To our knowledge, there has not been a study conducted specifically assessing the feasibility of replacing precontrast images with VUE images for evaluation of renal masses across a wide range of clinical scenarios for the rapid kVp‐switching DECT technique. The aim of this phantom study was to investigate the accuracy and sensitivity when measuring enhancement using VUE images across a variety of clinical conditions to assess the potential of replacing TUE images in diagnostic renal CT exams with VUE images derived from rapid‐kV‐switching DECT technology.

## 2 METHODS

**Figure 1:**
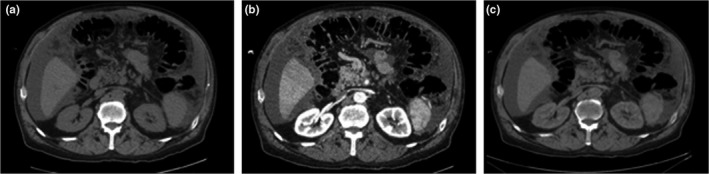
(a) True unenhanced image, (b) postcontrast image, (c) virtual unenhanced image reconstructed from Dual‐Energy computed tomography dataset.

The technique employed in the VUE image reconstruction is believed to utilize a two‐material decomposition technique, namely water and iodine.^8^ It can be assumed that iodine has displaced blood in postcontrast imaging; therefore, the amount of iodine estimated in each voxel can be replaced by an equivalent volume of blood to generate a VUE image (Fig. 1).^13^ This method of VUE image reconstruction is based on the assumption that materials within each voxel mix to form an ideal solution.

Phantoms were constructed and used to compare the accuracy of measured enhancement when VUE images were used as the baseline versus TUE images across a range of simulated clinical scenarios. Several variables known to affect measured CT number were evaluated. These variables included patient size, lesion size, Gemstone Spectral Imaging (GSI) protocol used, and level of simulated enhancement.

**Figure 2:**
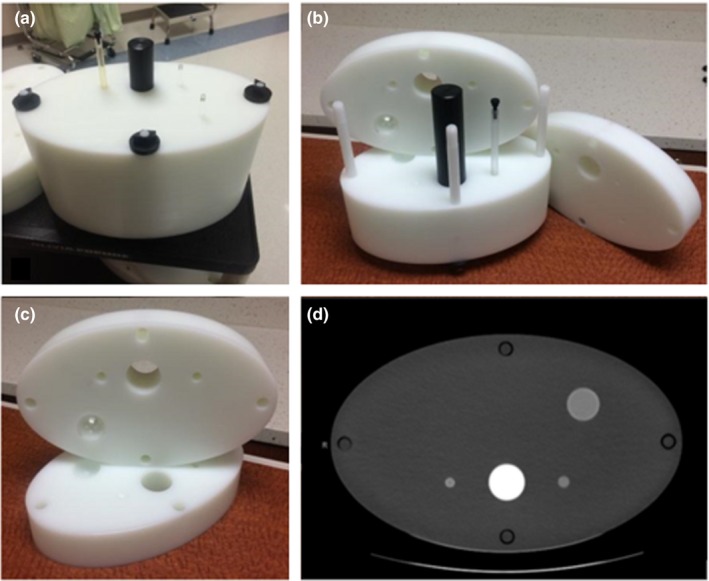
Small phantom used in this study: (a) fully assembled, (b) partially assembled, (c) a set of the two interior interchangeable plates, (d) computed tomography image of the phantom (axial view).

**Figure 3:**
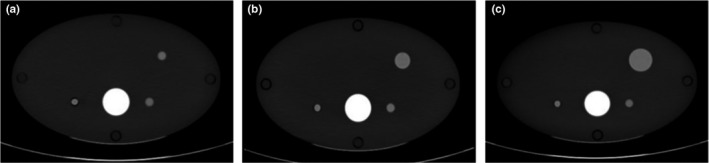
True unenhanced images of the small phantom demonstrating different spherical insert sizes in the periphery (a) 1.0 cm, (b) 2.0 cm, (c) 3.0 cm.

Three elliptical cylinder phantoms were designed and constructed for this study, referred to here as the small, medium, and large phantoms. Each phantom was composed of four plates made of high density polyethylene (Fig. 2). The major/minor axes of the phantoms were selected to correspond to the 5^th^ (28.3/17.4 cm), 50^th^ (36.1/22.2 cm), and 95^th^ (47.9/29.4 cm) percentiles of the adult population of the United States. Dimensions were calculated from the PeopleSize (Open Ergonomics, Ltd., Leicestershire, UK) anthropometric database. Lesion size and location were variable within each phantom, which was facilitated by fabricating three interchangeable sets of the two interior plates for each phantom. Using these plates, each phantom could contain a 1.0, 2.0, or 3.0 cm diameter spherical insert in the periphery. Additionally, the phantom included a 1.0‐cm spherical insert and a 1.0‐cm‐diameter cylindrical insert to the left and right of a Delrin rod, which was included to represent the spine (Fig. 3).

The phantom study was designed to simulate nonenhancing, intermediately enhancing, and highly enhancing renal lesions. A conceptual summary of all enhancement scenarios simulated is given in Table 1. Data for each scenario were acquired in the same general fashion using a single Discovery HD750 CT scanner (GE Healthcare, Waukesha, WI) in helical mode. Dual‐energy data processing and VUE image reconstruction was performed using version 2.0 of the GSI Volume Viewer (GE Healthcare, Waukesha, WI). For all simulations, the phantom was positioned identically for the pre‐ and postcontrast scans. In the postcontrast configuration, a single‐energy CT (SECT) scan was acquired at 120 kVp followed by the DECT dataset. The acquisition and reconstruction parameters used for both pre‐ and postcontrast CT data acquisition are provided in Table 2, where the mAs for each GSI protocol used in the study is specifically detailed. Note that the mAs used for dual energy acquisition is linked to the GSI protocol selected. VUE images were reconstructed from DECT datasets using the Material Suppressed Iodine (MSI) algorithm.

**Table 1:** Summary of clinical scenarios simulated in phantom study.

Scenario	TUE baseline (HU)	Enhancement (HU)	Purpose is to assess enhancement accuracy for a:
No enhancement	0	0	Simple benign cyst
Intermediate enhancement	20	10, 20	Mass that is borderline on TUE image and has borderline enhancement
	40	10, 20	Mass that is in 20‐70 HU “danger zone” [6] on TUE image and has borderline enhancement
Enhancement	40	40	A definitively enhancing mass

Abbreviations: HU, Hounsfield units; TUE, true unenhanced.

**Table 2:** Imaging parameters for the single phantom configuration used.

Parameter	DECT Technique	SECT Technique
Image thickness (mm)	5.0	5.0
SFOV (mm)	400	400
Pitch	0.984	0.984
kVp	80/140	120
mAs [GSI Protocol] (Est. CTDI_vol_)	480 [10, 11, 12] (25.5 mGy)	150 (14.1 mGy)
	384 [16] (22.9 mGy)	
	263 [22] (10.8 mGy)	
	440 [29] (27.6 mGy)	
	288 [36] (10.3 mGy)	

Abbreviations: DECT, Dual‐Energy CT; GSI, gemstone spectral imaging; SECT, single‐energy CT; SFOV, scan field of view.

Zero enhancement was simulated by imaging each phantom configuration with distilled water in the phantom’s inserts for pre‐ and postcontrast imaging. Intermediate enhancement was simulated by acquiring TUE images with a water‐blood surrogate mixture in each insert to achieve precontrast CT densities of 20 and 40 HU. These values were chosen because they corresponded to the lower bound and typical value for the known RCC “danger zone” of 20‐70 HU on precontrast imaging, which allows for simulation of borderline lesions.^6^ Note that apple juice was used as a blood surrogate in this study, as it was found to have a similar effective atomic number, density, and CT number to blood. Iodinated contrast (Optiray 320, GE Healthcare, Waukesha, WI) was added to each insert for postcontrast imaging to simulate 10 HU (low) and 20 HU (borderline) enhancement levels for each of the baseline precontrast values (Table 1).

Previous studies have found that the typical precontrast CT number of RCC is approximately 35‐40 HU,^20^ and that the known unenhanced CT number range for RCC is 20‐70 HU.^6^ Therefore, enhancing lesions were simulated by first acquiring TUE images with a water‐blood surrogate mixture to achieve a CT number of 40 HU. Enhancement was simulated by adding iodinated contrast to achieve a postcontrast CT number of approximately 80 HU in the spherical simulated lesion inserts. This value was chosen because enhancement of 42 HU or more has been shown to be highly predictive of RCC.^7^

An empirical relationship between CT number (HU) at 120 kVp and iodinated contrast concentration (mg/mL) was calculated and used to determine the contrast needed to achieve each desired enhancement. DECT GSI protocols were selected to evaluate the widest range of acquisition variables possible. The selection of GSI protocols was informed by a previous experiment which compared the CT numbers measured in VUE image for all GSI protocols to the CT number measured in the TUE image, which was considered the ground truth. A subset of four GSI protocols were selected for this experiment. The GSI 10 protocol was used for all phantom sizes to allow for a direct comparison of the effect of phantom size on measured enhancement for a given technique. VUE images were reconstructed from the DECT dataset using the MSI algorithm available on the GSI volume viewer. Measured enhancement was quantified for the SECT dataset by calculating the difference in measured CT number between the 120 kVp postcontrast scan and the TUE images (henceforth referred to as ΔTUE). Similarly, enhancement was calculated for the DECT dataset as the difference in CT number between 70‐keV monochromatic images processed from the DECT postcontrast dataset and the VUE images (henceforth referred to as ΔVUE). The sizes of ROIs used to measure enhancement were identical in the pre‐ and postcontrast images and across experiments. The ROI size used was 14.4 mm^2^ (1‐cm sphere and hollow rod), 70‐80 mm^2^ (2‐cm insert), and 140‐150 mm^2^ (3‐cm insert). Each ROI was placed in the center of the insert on the central slice of the phantom. Variation between ROI measurement locations was considered as a source of random error in the statistical model. All images were reconstructed at an image thickness of 5.0 mm using the STANDARD kernel with no iterative reconstruction applied.

Data were analyzed using Bayesian hierarchical models that incorporated all sources of experimental uncertainty. In each model, the Bayesian method was used to estimate the parameters of the posterior distribution in multiple levels, then combined to form the hierarchical model. This method allowed for accounting of all sources of uncertainty in the study. The result of this analysis was the posterior probability. Separate models were built for nonenhancing, intermediate enhancing, and positively enhancing simulated lesions. Sources of random error incorporated into the model included CT scanner variability (σ_S_), variation between ROI measurement location within the simulated lesion (σ_M_), and variation across the ROI, or noise (σ_N_). For the experimental scenarios, all images needed for each phantom configuration were acquired using a single preparation of the solution used to fill the inserts. A 2‐mL serological pipette with a specified precision of ± 0.01 mL (Fisherbrand, Fisher Scientific, Pittsburgh, PA) was used to pipette the Optiray 320. The total experimental variation was calculated by adding each source of variation in quadrature for each measurement. The fixed effects incorporated in the model were lesion size, lesion location, phantom size, enhancement, and GSI protocol used.

A posterior probability > 0.95 for the difference between ΔVUE and ΔTUE returned by the Bayesian hierarchical model indicated that ΔVUE was significantly higher than ΔTUE enhancement measurements, while a posterior probability < 0.05 indicated that ΔVUE was significantly lower than ΔTUE. A posterior probability of 0.05‐0.95 indicated no significant difference between ΔVUE and ΔTUE. Note that the credible interval (CI) is a range within which lies some predetermined percentage (e.g., 95%) of the posterior distribution of the parameters given the data and can be interpreted as the Bayesian analogue of the confidence interval.

## 3 RESULTS

### 3.1 No enhancement

**Figure 4:**
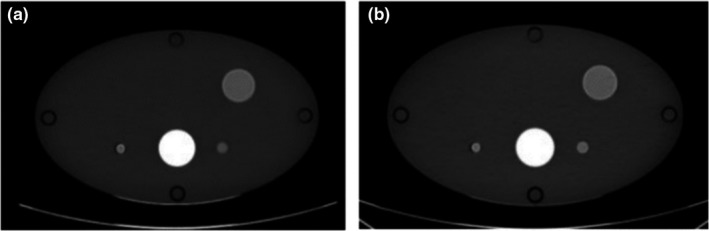
Example of images used for measuring enhancement in simple nonenhancing lesions (a) true unenhanced image (b) virtual unenhanced image reconstructed from Dual‐Energy computed tomography dataset.

For simulations of simple nonenhancing lesions, CT numbers were directly compared between TUE and VUE images (Fig. 4). Measured CT numbers matched well between VUE and TUE images (Table 3), and in all cases, the precontrast CT number measured in VUE images was not significantly different from that measured in TUE images. Measured CT numbers were lower in VUE than TUE images for the large phantom, but the difference was not significant.

**Table 3:** Posterior probabilities as calculated using the Bayesian hierarchical model for simulation of nonenhancing lesions.

GSI protocol used	Small phantom		Medium phantom		GSI protocol used	Large phantom	
	95% CrI^a^ for difference	Posterior probability	95% CrI for difference	Posterior probability		95% CrI for difference	Posterior probability
10	(−1.5, 2.4)	0.564	(−2.1, 1.8)	0.502	10	(−10.2, 1.0)	0.238
11	(−1.3, 2.4)	0.555	(−1.8, 2.2)	0.515	12	(−10.0, 1.0)	0.250
16	(−1.4, 2.7)	0.560	(−1.3, 3.0)	0.560	22	(−11.1, 0.9)	0.226
29	(−1.6, 2.1)	0.524	(−1.6, 2.4)	0.531	36	(−8.9, 1.4)	0.273
σ_Protocol_ b	(0.029, 2.4)		(0.029, 2.6)			(0.037, 7.3)	

Abbreviation: GSI, gemstone spectral imaging.

a CrI = credible interval for difference in measured computed tomography number between virtual unenhanced and true unenhanced imaging.

b Represents the total random error used as input to Bayesian hierarchical model.

### 3.2 Intermediate enhancement

**Figure 5:**
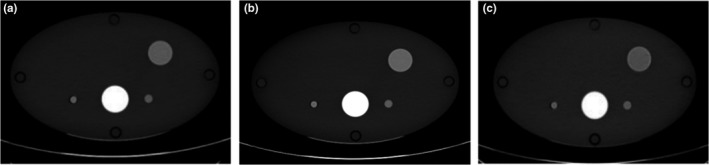
Example of images used for measuring enhancement in intermediately enhancing lesions (a) true unenhanced image (b) 70‐keV monochromatic image reconstructed from the Dual‐Energy post‐contrast dataset (c) virtual unenhanced image reconstructed from Dual‐Energy computed tomography dataset.

ΔVUE was lower than ΔTUE for intermediate enhancing, or borderline, simulated lesions (Fig. 5). A posterior probability of < 0.05 was returned by the Bayesian hierarchical model for all intermediate scenarios, indicating that ΔVUE was significantly lower than ΔTUE (Table 4). The difference in measured enhancement decreased with increasing phantom size. The effect of patient size on measured enhancement can be seen by examining the 95% credible interval (CI) for each phantom size.

**Table 4:** The 95% credible intervals for the difference between ΔVUE‐ and ΔTUE‐based enhancement measurements as calculated using a Bayesian hierarchical model for simulation of intermediate enhancing lesions. Note that the posterior probability for all protocols and phantom sizes was < 0.05.

GSI protocol used	Small phantom	Medium phantom	GSI protocol used	Large phantom
	95% CrI^a^ for difference	95% CrI for difference		95% CrI for difference
10	(−18.3, −14.5)	(−15.6, −12.2)	10	(−10.3, −4.6)
11	(−18.3, −14.6)	(−15.9, −12.6)	12	(−9.7, −3.9)
16	(−18.5, −14.6)	(−15.8, −12.4)	22	(−11.3, −5.9)
29	(−18.1, −14.4)	(−16.0, −12.6)	36	(−10.2, −4.6)
σ_Protocol_ b	(4.36, 21.2)	(3.76, 18.3)		(1.95, 10.5)

Abbreviations: GSI, gemstone spectral imaging; TUE, true unenhanced; VUE, virtual unenhanced.

a CrI = credible interval for difference in measured CT number between ΔVUE and ΔTUE imaging.

b Represents the total random error used as input to Bayesian hierarchical model.

### 3.3 Positive enhancement

**Figure 6:**
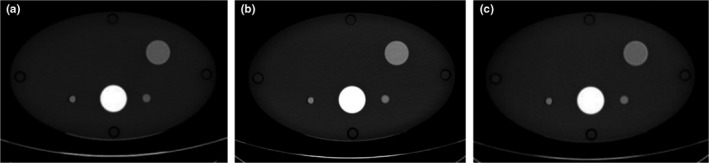
Example of images used for measuring enhancement in positively enhancing lesions (a) true unenhanced image (b) 70‐keV monochromatic image reconstructed from the Dual‐Energy post‐contrast dataset (c) virtual unenhanced image reconstructed from Dual‐Energy computed tomography dataset.

ΔVUE was significantly less than ΔTUE for simulation of positively enhancing lesions (Fig. 6 and Table 5), which is similar to the results for simulation of intermediate lesions. However, there was no trend in the enhancement differences, which were of similar magnitude independent of phantom size and GSI protocol.

**Table 5:** The 95% credible intervals for the difference between ΔVUE‐ and ΔTUE‐based enhancement measurements as calculated using a Bayesian hierarchical model for simulation of enhancing lesions. Note that the posterior probability for all protocols and phantom sizes was < 0.05.

GSI protocol used	Small phantom	Medium phantom	GSI protocol used	Large phantom
	95% CrI^a^ for difference	95% CrI for difference		95% CrI for difference
10	(−10.5, −5.9)	(−12.0, −5.8)	10	(−13.2, −6.2)
11	(−12.1, −7.3)	(−13.1, −6.7)	12	(−11.2, −4.6)
16	(−11.8, −7.0)	(−12.9, −6.2)	22	(−14.3, −7.3)
29	(−11.4, −6.5)	(−12.2, −5.5)	36	(−11.6, −5.0)
σ_Protocol_ b	(2.39, 12.27)	(2.40, 12.67)		(2.38, 12.86)

Abbreviation: GSI, gemstone spectral imaging.

a CrI = credible interval for difference in measured CT number between ΔVUE and ΔTUE imaging.

b Represents the total random error used as input to Bayesian hierarchical model.

### 3.4 Effect of lesion size on measured enhancement

A Bayesian hierarchical model was used to calculate the standard deviation of enhancement, σ_lesion_, resulting from variation in simulated lesion size (Table 6). σ_lesion_ was calculated for the 1.0‐cm and 3.0‐cm simulated lesions relative to the enhancement measured in the 2.0‐cm simulated lesion. A posterior probability > 0.95 indicated that the measured enhancement was significantly higher than for the 2.0‐cm simulated lesion, while a posterior probability < 0.05 indicated the measured enhancement was significantly lower. Significant differences were observed between enhancement measurements in the 1.0‐and 2.0‐cm simulated lesions for positive enhancement in the medium phantom. No other significant differences were noted.

**Table 6:** Bayesian hierarchical model output for effect of simulated lesion size on measured enhancement. Calculations were made for the 1‐ and 3‐cm simulated lesion sizes relative to the 2‐cm lesion size.

Enhancement scenario	Lesion size (cm)	Small phantom		Medium phantom		Large phantom	
		95% CrI^a^ for difference (σ_lesion_)	Posterior probability	95% CrI for difference (σ_lesion_)	Posterior probability	95% CrI for difference (σ_lesion_)	Posterior probability
Intermediate	1.0	(−2.13, 0.86)	0.197	(−2.30, 0.77)	0.154	(−5.19, 0.82)	0.074
	3.0	(−0.39, 2.48)	0.928	(−0.73, 2.10)	0.849	(−2.49, 3.12)	0.587
Positive	1.0	(−4.08, 1.04)	0.127	(−6.76, 0.02)	0.026	(−4.90, 1.39)	0.138
	3.0	(−0.67, 4.24)	0.922	(−0.99, 5.91)	0.921	(−1.56, 4.16)	0.828

a CrI = credible interval for difference.

Sensitivity, specificity, and accuracy were calculated for the entire phantom study, across all simulations. For this computation, all enhancement values < 15 HU were considered to be negative for enhancement, and all values ≥ 15 HU were considered positive for enhancement. The sensitivity was 43.2%, specificity was 100%, and accuracy was 78.1% for all enhancement scenarios with any amount of iodine present. All enhancement measurements categorized as negative using ΔTUE were also categorized as negative using ΔVUE. This is reflected in the results of the Bayesian hierarchical models, where all significant differences (<0.05) resulted from ΔVUE measurements being significantly lower than ΔTUE measurements. Clinically, this implies that nonenhancing masses can be accurately categorized using VUE images in place of TUE images. A sensitivity of 43.2% indicates that VUE images may not be a suitable replacement for TUE images for identification of masses with unknown levels of enhancement.

## 4 DISCUSSION

The simulation of nonenhancing lesions provides a baseline comparison of VUE to TUE images. Considering the fundamental principles of MSI image reconstruction, it is expected that in the absence of iodine signal there would be no significant differences between CT numbers measured in VUE and TUE images. The results of this simulation indicated that there were no significant differences between measured CT numbers in VUE and TUE images for nonenhancing lesions, as expected.

The quantification of enhancement has been stated to be the most important criterion in distinguishing surgical from nonsurgical masses,^2^ therefore the accurate quantification of enhancement in borderline masses is of particular importance. The posterior probabilities calculated from the Bayesian hierarchical model for simulation of intermediate enhancing masses were < 0.05 for all GSI protocols and phantom sizes (Table 4), indicating that ΔVUE enhancement measurements were significantly lower than ΔTUE enhancement measurements. This result has clinical significance, because it is for intermediate and low enhancing masses that the accurate quantification of enhancement is most important. The impact of these results is consistent with a previous study that retrospectively compared CT numbers on TUE versus VUE images directly and found a consistent difference between the images of 5‐9 HU across seven anatomical locations.^23^ A difference of 5‐9 HU is enough to cause incorrect characterization of a borderline lesion as nonenhancing, which was demonstrated in this study by a sensitivity of 43.2%.

For highly enhancing masses, the Bayesian hierarchical model returned posterior probabilities < 0.05 for all phantom configurations, indicating that ΔVUE measurements were significantly lower than ΔTUE measurements. However, the sensitivity for correct categorization of these masses as enhancing was 91.2% for ΔVUE measurements. This may indicate that the difference between enhancement quantified using ΔVUE and ΔTUE is not clinically significant for highly enhancing masses. This experiment has demonstrated that for highly enhancing renal lesions, enhancement calculations for VUE and TUE images would lead to the same categorization of the lesion, and therefore confirm that VUE images would be a feasible replacement to TUE images in these cases. However, given the results of the intermediate enhancement experiment, the overall results of this study indicate that it may not be feasible to replace TUE images with VUE images as a baseline for calculating enhancement.

During multiphase renal CT, the renal parenchyma has been shown to enhance by as much as 145 to 185 HU,^21^ from 30‐40 HU on precontrast imaging to upward of 250 HU in the corticomedullary phase. This can result in pseudoenhancement of small lesions^22^ and is an important consideration when using VUE images, which are generated from postcontrast data sets. The phantoms used in this study could not be configured to have background enhancement beyond that of the simulated lesions, as is observed during the corticomedullary phase of renal CT. To investigate the potential effects of pseudoenhancement, the Jascszak phantom (Data Spectrum Corporation, Durham, NC) was imaged in two configurations. In both configurations, the spherical inserts were filled with an Optiray 320‐water mixture to achieve a CT number of approximately 100 HU. In one configuration, the background was filled with water only, and in the other configuration the background was filled with an Optiray 320‐water mixture to achieve a CT number of approximately 230 HU to simulate strongly enhancing renal parenchyma. CT numbers were measured in the 25.4‐mm sphere on SECT images, 70‐keV monochromatic images, and VUE images for both phantom configurations at an image thickness of 2.5 mm. The observed pseudoenhancement was 17 HU for SECT and 24 HU for 70‐keV monochromatic images. The measurements made in VUE images at the same location had identical CT numbers (15 HU) for both phantom configurations. The measured level of pseudoenhancement is consistent with that previously reported for SECT,^24^ while higher levels were observed for 70‐keV monochromatic images. The pseudoenhancement observed in the 70‐keV monochromatic images did not affect the corresponding VUE images. This may be related to the DECT processing workflow used, which in the implementation studied in this work, occurs in the projection domain. In any case, the observed differences in CT number resulting from pseudoenhancement would tend only to *increase* measured enhancement slightly, consistent with pseudoenhancement. This is opposite the effect we observed when replacing TUE images with VUE images, where incomplete removal of iodine signal resulted in significantly *decreased* measured enhancement.

For completeness, it is important to note potential differences in CT numbers measured in 120‐kVp SECT and 70‐keV images, which were reconstructed from a DECT dataset acquired at 80 and 140 kVp. An experiment was conducted to compare CT numbers measured in monochromatic images reconstructed from the same DECT dataset with energy ranging from 65 to 75 keV to assess the impact of choice of energy for postcontrast images on measured enhancement. The maximum difference between the CT number measured in 70‐keV images and any energy in the range 65‐75 keV was 3.7 HU. This implies a maximum increase of 3.7 HU in measured enhancement, which still resulted in measured enhancement being significantly lower for ΔVUE compared to ΔTUE for all simulations. This indicates that the largest contributor to differences in measured enhancement resulted from the use of VUE images as the precontrast baseline.

Limitations of this study include the use of a blood surrogate as opposed to the use of blood. The MSI algorithm has been stated to replace the estimated volume of iodine with an equivalent volume of blood.^13^ In light of this, the use of blood for TUE baseline image acquisition and blood/Optiray mixtures would have been the most preferable experimental approach. However, due to limitations regarding the accessibility of blood a surrogate material was used, which may have affected the results of this study. Additionally, the phantom background was made of high density polyethylene, which has a spectral curve that is different from that of soft tissue. The CT number of the phantom background was measured to be approximately −65 HU at 120 kVp, which is lower than the CT number of soft tissue and abdominal organs.

Future work includes further phantom study with blood or investigation of material suppression techniques from other DECT technologies (e.g., dual‐source DECT). An additional study should be conducted to determine the optimal postcontrast phase for VUE image reconstruction. Further work could include a more thorough investigation to determine the optimal GSI protocol for VUE image reconstruction that would most closely match the corresponding TUE image.

## 5 CONCLUSIONS

In this phantom study, VUE images provided an accurate baseline for enhancement classification of nonenhancing cysts. Although ΔVUE was significantly less than ΔTUE for highly enhancing lesions, the measured enhancement for ΔVUE was still high enough that the measured enhancement would lead to the same clinical conclusion for positively enhancing lesions (>40 HU). However, VUE images did not provide an accurate baseline for enhancement quantification of intermediate or borderline enhancing lesions and could result in the classification of a mass with low or borderline enhancement as nonenhancing. As a result, we conclude that VUE images may not be a suitable replacement for TUE images for the task of measuring enhancement of renal masses, as the results of this study do not support the hypothesis that VUE images can replace TUE images for enhancement quantification in renal masses with this version of the MSI algorithm.

## CONFLICTS OF INTEREST

The authors have no relevant conflicts of interest to disclose.
